# Supplementary material for: DeepSynBa: actionable drug combination prediction with complete dose-response profiles
Source: Bioinformatics. 2026 Jun 20;42(7):btag416. doi: 10.1093/bioinformatics/btag416 (PMC13341016; doi:10.1093/bioinformatics/btag416)
Supplement: btag416_Supplementary_Data [file btag416_supplementary_data.pdf]

Supplementary Information for DeepSynBa: Actionable Drug Combination Prediction with Complete Dose-Response Profiles

1 Tables

	RMSE	Pearson	Spearman
**DeepSynBa ∼ comboFM**	4.78×10^{-24}	6.52×10^{-33}	8.18×10^{-23}
**DeepSynBa ∼ comboLTR**	3.51×10^{-34}	1.26×10^{-30}	5.60×10^{-31}
**DeepSynBa ∼ comboKR 2.0**	7.31×10^{-48}	2.37×10^{-66}	3.71×10^{-58}
**DeepSynBa ∼ DD-PRISM**	1.47×10^{-30}	6.66×10^{-32}	3.27×10^{-22}
**comboFM ∼ comboLTR**	4.35×10^{-10}	6.19×10^{-18}	1.08×10^{-6}
**comboFM ∼ comboKR 2.0**	1.04×10^{-37}	3.79×10^{-69}	6.37×10^{-29}
**comboFM ∼ DD-PRISM**	1.59×10^{-1}	7.92×10^{-2}	7.44×10^{-1}
**comboLTR ∼ comboKR 2.0**	1.04×10^{-24}	5.82×10^{-21}	3.29×10^{-13}
**comboLTR ∼ DD-PRISM**	5.00×10^{-8}	1.16×10^{-15}	6.81×10^{-6}
**comboKR 2.0 ∼ DD-PRISM**	8.45×10^{-36}	1.74×10^{-64}	8.83×10^{-27}

Table S1: The pairwise t-test p-value results for cell line based results in NCI-ALMANAC.

	**RMSE**	**Pearson**	**Spearman**
**DeepSynBa ∼ comboFM**	6.11×10^{-38}	4.17×10^{-33}	4.38×10^{-14}
**DeepSynBa ∼ comboLTR**	1.39×10^{-55}	4.63×10^{-58}	2.29×10^{-25}
**DeepSynBa ∼ comboKR 2.0**	4.66×10^{-37}	1.23×10^{-44}	6.58×10^{-42}
**DeepSynBa ∼ DD-PRISM**	8.85×10^{-33}	5.98×10^{-26}	3.15×10^{-13}
**comboFM ∼ comboLTR**	2.26×10^{-15}	1.41×10^{-25}	4.52×10^{-4}
**comboFM ∼ comboKR 2.0**	1.30×10^{-23}	3.48×10^{-30}	2.06×10^{-13}
**comboFM ∼ DD-PRISM**	2.73×10^{-1}	5.41×10^{-1}	9.68×10^{-1}
**comboLTR ∼ comboKR 2.0**	1.05×10^{-13}	2.73×10^{-10}	5.11×10^{-5}
**comboLTR ∼ DD-PRISM**	2.09×10^{-10}	7.52×10^{-20}	7.91×10^{-4}
**comboKR 2.0 ∼ DD-PRISM**	3.8×10^{-22}	1.67×10^{-28}	1.52×10^{-12}

Table S2: The pairwise t-test p-value results for drug based results in NCI-ALMANAC.

	**Hyperparameter**	**Values**
Drug-Cell Encoder	hidden layers	$256, 512, 1024, 2048, 4096$
	activations	ReLU
	input dropout	none, 0.2, $0.3, 0.5$
	dropout	$0.2, 0.5$
	normalizations	batch normalization
Prediction heads	hidden layers	$64, 128, 256$
	activations	ReLU
	final layer activation	ReLU, sigmoid, linear
	normalizations	layer normalization
Training parameter	number of epochs	500
	loss	MSE, L1
	scheduler	LinearLR
	optimizer	AdamW (lr=10^{-4}, wc=10^{-5})
	batch size	128

Table S3: The search space for tuning hyperparameters and training parameters for DeepSynBa architecture.

	**MolFormer**	**ECFP4**
**RMSE**	**7.72**	**7.37**
**Pearson**	**0.95**	**0.96**
**Spearman**	**0.82**	**0.83**

Table S4: Comparison of model performance across different drug representations. MolFormer uses transformer-based MolFormer model to extract drug features while ECFP4 is the drug fingerprints.

	**GEX only**	**GEX + MUT + CNV + METHY**
**RMSE**	7.72	8.66
**Pearson**	0.95	0.94
**Spearman**	0.82	0.78

Table S5: Comparison of model performance across different cell line representations. The GEX-only model relies solely on gene expression profiles, while the GEX + MUT + CNV + METHY model integrates additional genomic features, including mutations, copy number variations, and DNA methylation.

	DeepSynBa	Matrix Prediction Head
RMSE	**7.72**	**22.57**
Pearson	**0.95**	**0.69**
Spearman	**0.82**	**0.54**

Table S6: Comparison of alternative prediction head that directly outputs dose–response matrices. Instead of predicting SynBa parameters and reconstructing responses via the likelihood formulation, the model predicts a 4 × 4 response matrix R_i for each drug in the combination. The final dose–response matrix is obtained by integrating these predicted matrices with the corresponding drug dosages.

2 Figures

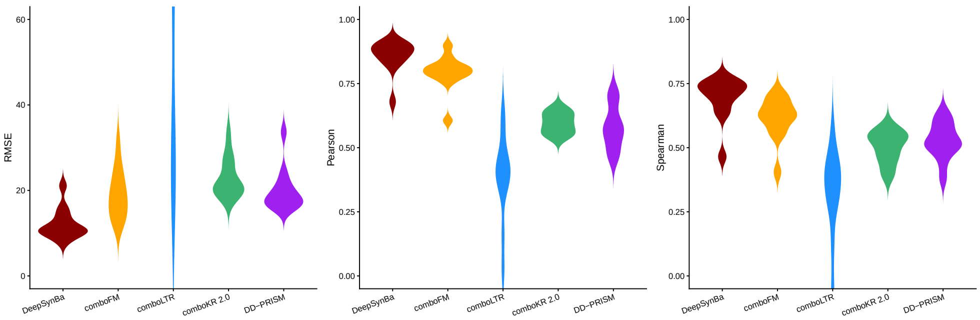

(a) Cell line based results on new cell scenario.

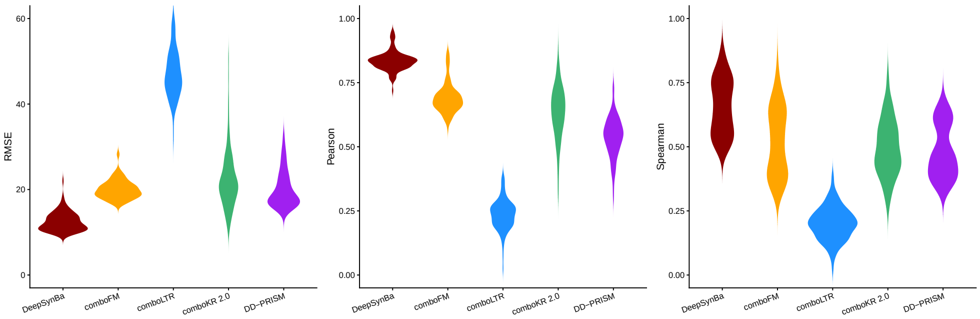

(b) Drug based results on new cell scenario.

Figure S1: The distributions of RMSE, Pearson and Spearman correlations of dose-response surface predictions on different (a) cell line and (b) drugs with new cell scenario in NCI-ALMANAC.

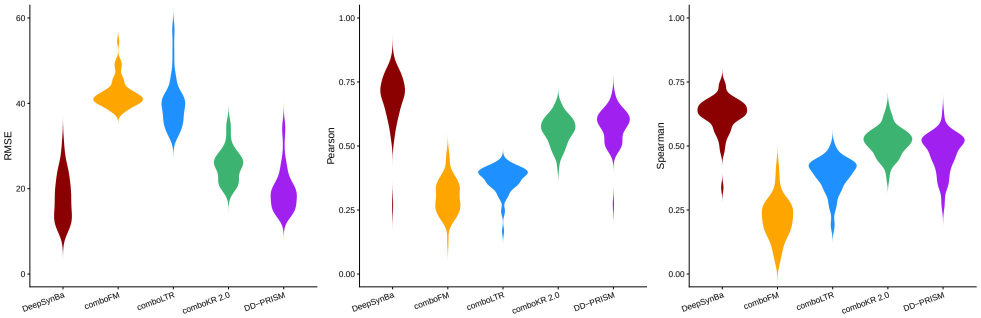

(a) Cell line based results on new drug scenario.

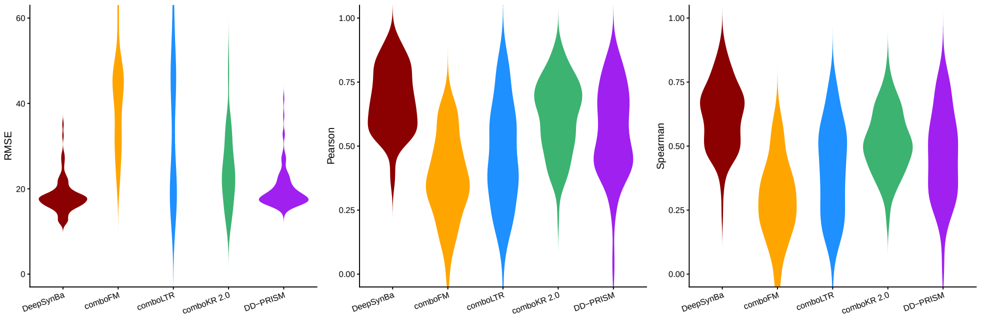

(b) Drug based results on new drug scenario.

Figure S2: The distributions of RMSE, Pearson and Spearman correlations of dose-response surface predictions on different (a) cell line and (b) drugs with new drug scenario in NCI-ALMANAC.

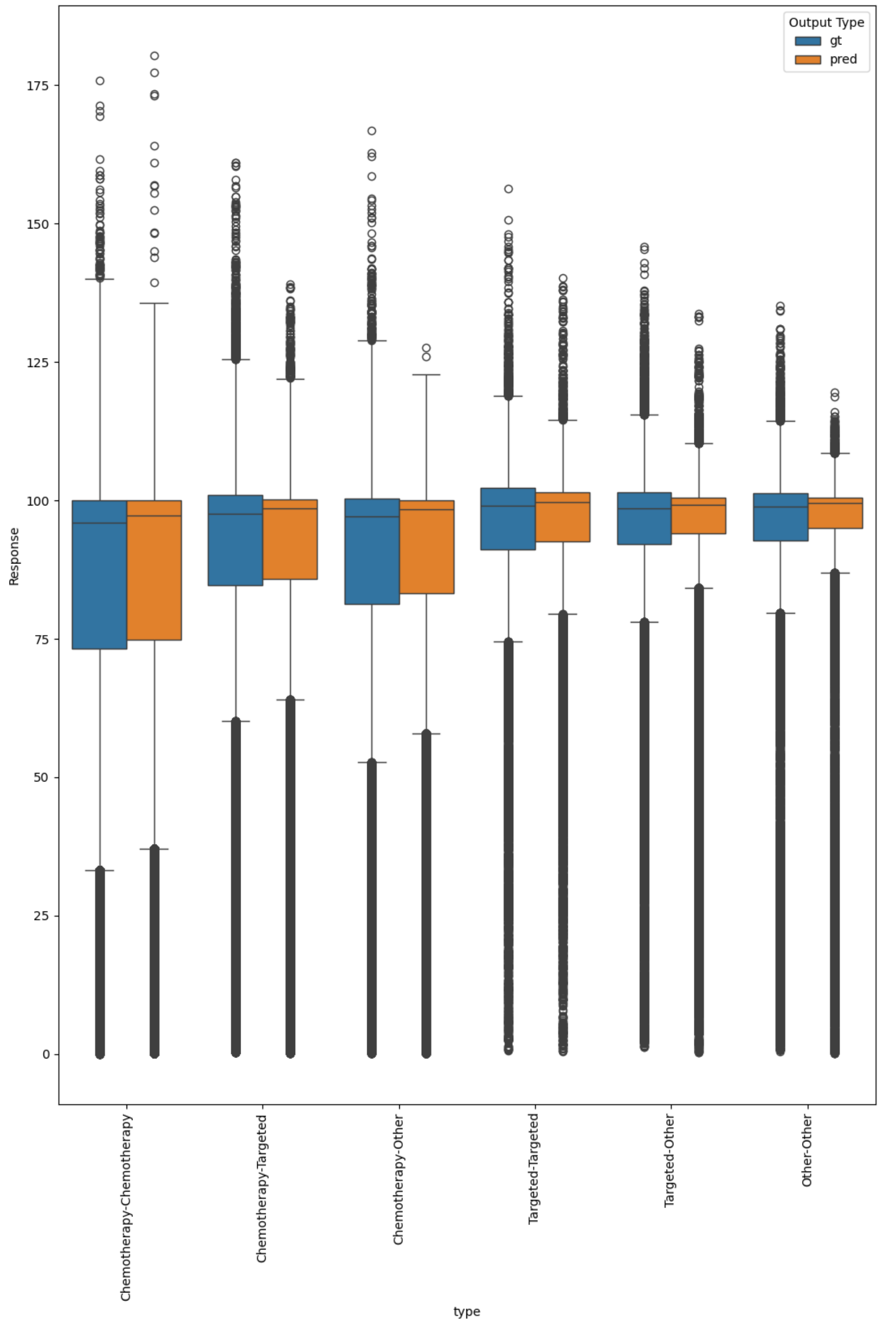

Figure S3: The ground-truth and the predicted response distributions for drug types in the combination for NCI-ALMANAC.

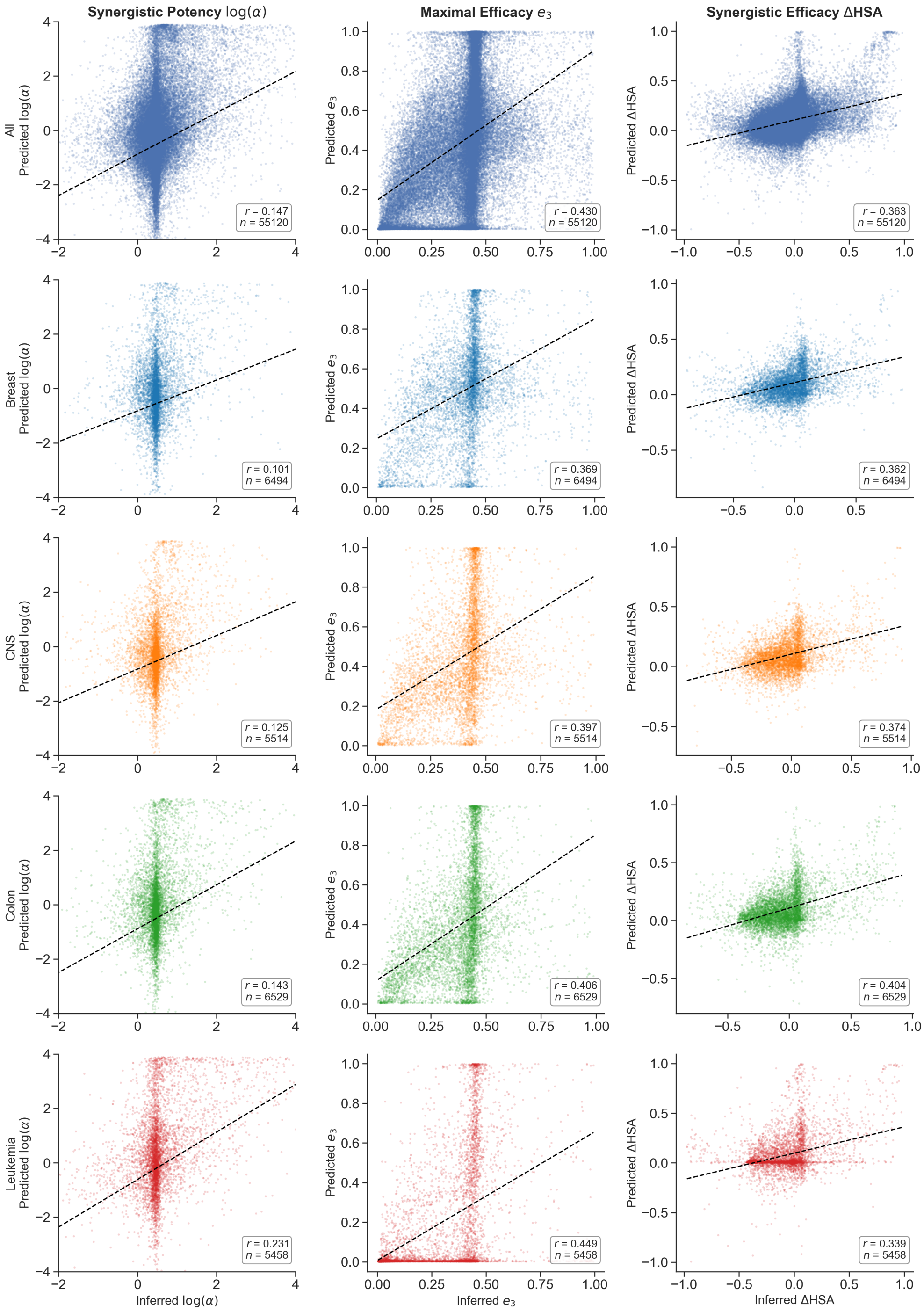

Figure S4: Tissue-stratified comparison of estimated and predicted synergy parameters in Scenario (i) (unseen drug combinations) of NCI-ALMANAC. The first row includes all cell types. Each subsequent row corresponds to an individual cancer tissue type. All rows show the same three metrics, i.e. (left) the synergistic potency ($\log \alpha$), (middle) the maximal efficacy (e_3), and (right) the synergistic efficacy ($\Delta\text{HSA} = \min\{e_1, e_2\} - e_3$). Pearson correlation coefficient r and sample size n are reported per panel. Dashed lines denote ordinary least-squares fits.

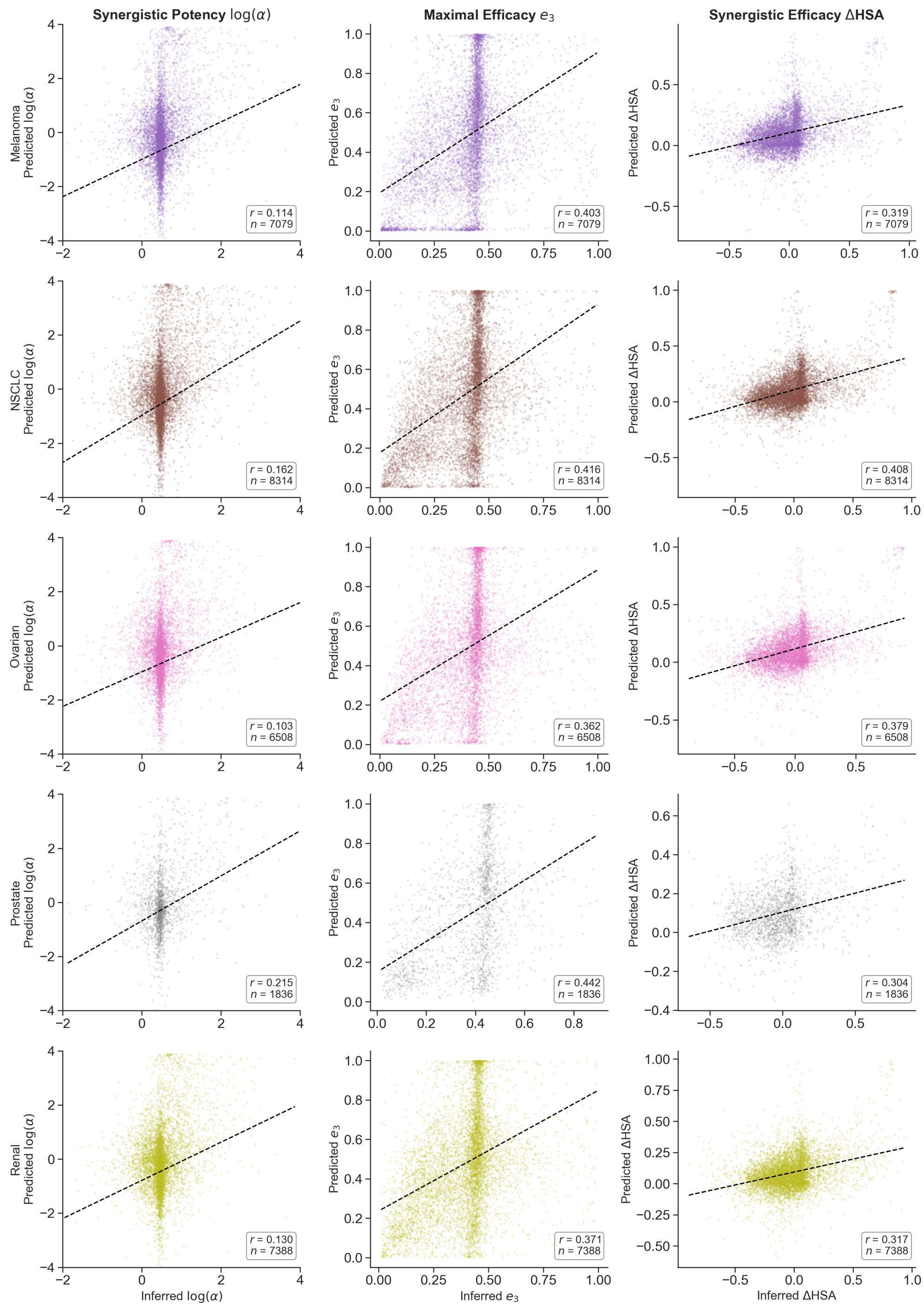

Figure S4: (Continued)

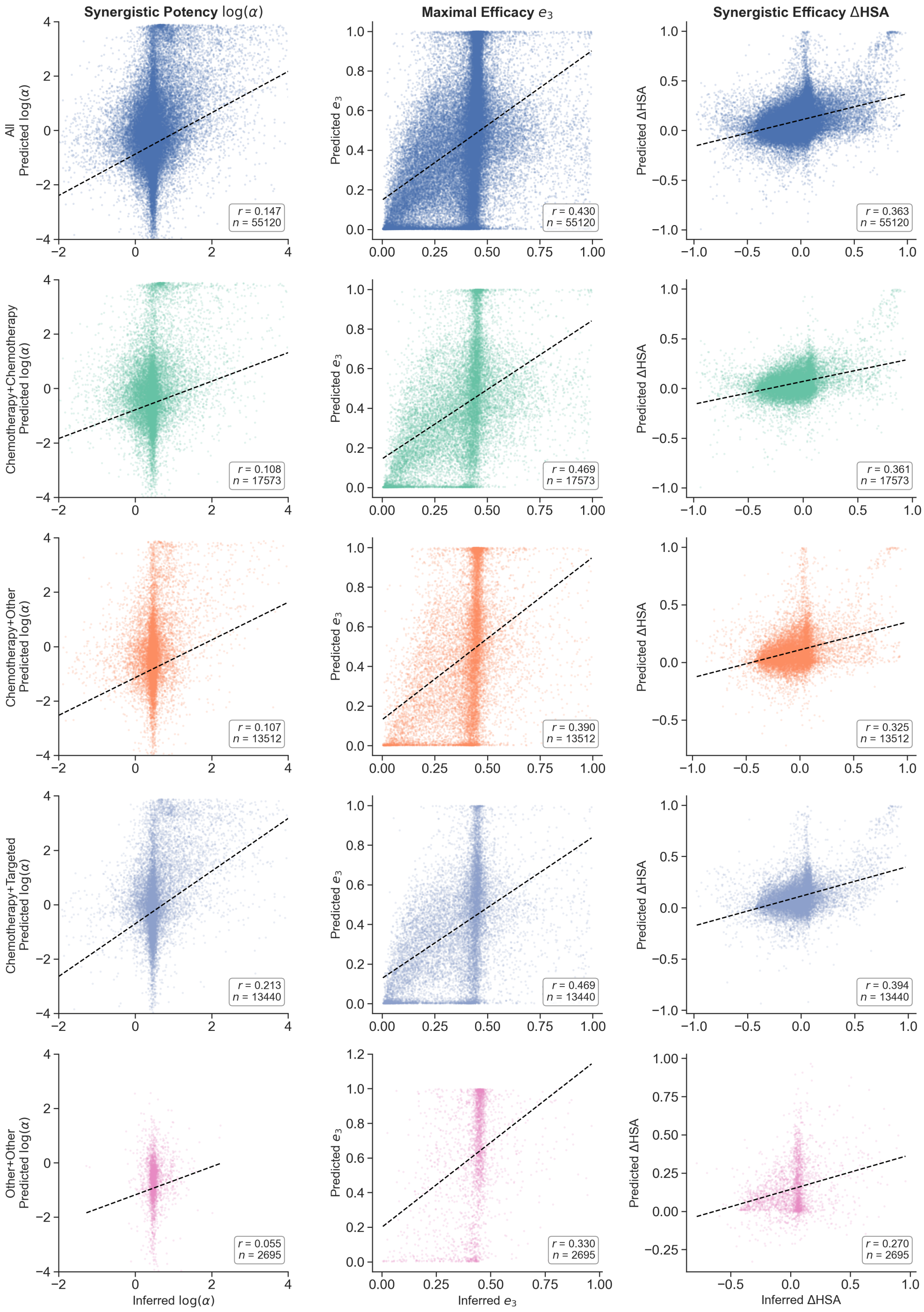

Figure S5: Drug-type-stratified comparison of estimated and predicted synergy parameters in Scenario (i) (unseen drug combinations) of NCI-ALMANAC. The first row includes all cell types. Each subsequent row corresponds to a pairwise combination of drugs from distinct therapeutic classes. All rows show the same three metrics as Fig. S4. Pearson correlation coefficient r and sample size n are reported per panel. Dashed lines denote ordinary least-squares fits.

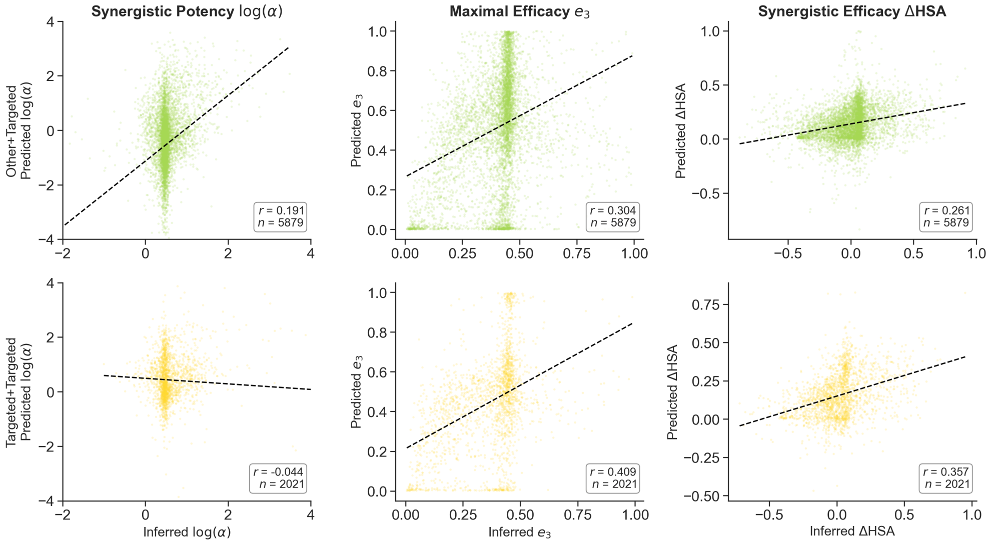

Figure S5: (Continued)

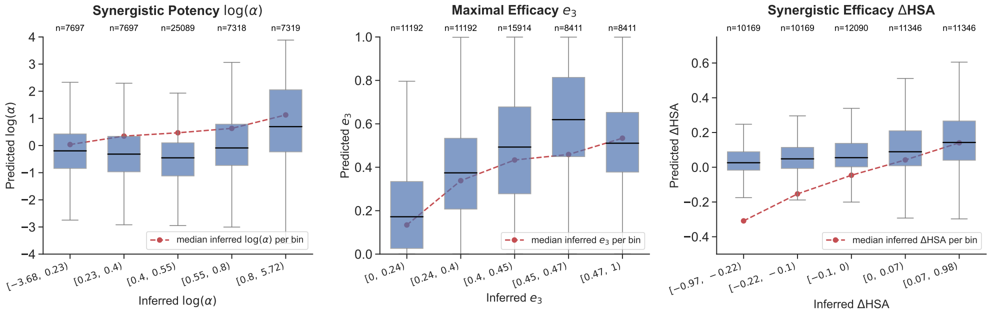

Figure S6: Distribution of predicted parameter values stratified by inferred value, for (left) the synergistic potency ($\log\alpha$), (middle) the maximal efficacy (e_3), and (right) the synergistic efficacy ($\Delta\text{HSA} = \min\{e_1, e_2\} - e_3$). For each parameter, combinations are partitioned into five bins along the inferred-value axis. The bin construction is identical across panels, consisting of a narrow central bin spanning the modal region of the inferred distribution (bin 3), and two outer bins on each side whose split points are the medians of the below-central and above-central populations, ensuring that bins 1 and 2 contain equal counts, as well as bins 4 and 5. Within each bin, the distribution of predicted values is summarised as a boxplot. The red dashed line connects, at each bin, the median of the inferred values falling in that bin. Under a perfectly calibrated model, the black median bar of each box would coincide with this marker. Sample sizes n are annotated above each box.
